# Epinecidin-1 Protects against Methicillin Resistant *Staphylococcus aureus* Infection and Sepsis in Pyemia Pigs

**DOI:** 10.3390/md17120693

**Published:** 2019-12-09

**Authors:** Han-Ning Huang, Chieh-Yu Pan, Bor-Chyuan Su, Hung-Yi Wu, Jyh-Yih Chen

**Affiliations:** 1 Marine Research Station, Institute of Cellular and Organismic Biology, Academia Sinica, 23-10 Dahuen Road, Jiaushi, Ilan 262, Taiwan; henryhw0125@gmail.com; 2 Department and Graduate Institute of Aquaculture, National Kaohsiung University of Science and Technology, Kaohsiung 811, Taiwan; panjade@nkust.edu.tw; 3 Department of Anatomy and Cell Biology, School of Medicine, College of Medicine, Taipei Medical University, Taipei 110, Taiwan; su8265@gmail.com; 4 Department of Veterinary Medicine, College of Veterinary Medicine, National Pingtung University of Science and Technology, Pingtung 91201, Taiwan

**Keywords:** antimicrobial peptide (AMP), epinecidin-1, MRSA, pyemia pigs

## Abstract

Methicillin resistant *Staphylococcus aureus* (MRSA) may be found on the skin, nose, and throats of long-term hospitalized patients. While MRSA infections are usually minor, serious infections and death may occur in immunocompromised or diabetic patients, or after exposure of MRSA to blood. This report demonstrates that the antimicrobial peptide (AMP) epinecidin-1 (Epi-1) efficiently protects against MRSA infection in a pyemia pig model. We first found that Epi-1 exhibits bactericidal activity against MRSA. Next, pharmacokinetic analysis revealed that Epi-1 was stable in serum for 4 h after injection, followed by a gradual decrease. This pharmacokinetic profile suggested Epi-1 may bind serum albumin, which was confirmed in vitro. Harmful effects were not observed for doses up to 100 mg/kg body weight in pigs. When Epi-1 was supplied as a curative agent 30 min post-infection, MRSA-induced abnormalities in blood uric acid (UA), blood urea nitrogen (BUN), creatine (CRE), GOT, and GPT levels were restored to normal levels. We further showed that the bactericidal activity of Epi-1 was higher than that of the antibiotic drug vancomycin. Epi-1 significantly decreased MRSA counts in the blood, liver, kidney, heart, and lungs of infected pigs. Elevated levels of serum C reactive protein (CRP), proinflammatory cytokine IL6, IL1β, and TNFα were also attenuated by Epi-1 treatment. Moreover, the MRSA genes, enterotoxin (et)-A, et-B, intrinsic methicillin resistance A (mecA), and methicillin resistance factor A (femA), were significantly reduced or abolished in MRSA-infected pigs after treatment with Epi-1. Hematoxylin and eosin staining of heart, liver, lung, and kidney sections indicated that Epi-1 attenuated MRSA toxicity in infected pigs. A survival study showed that the pyemia pigs infected with MRSA alone died within a week, whereas the pigs post-treated with 2.5 mg/kg Epi-1 were completely protected against death. The present investigation, thus, demonstrates that Epi-1 effectively protects pyemia pigs against pathogenic MRSA without major toxic side effects.

## 1. Introduction

Infection by the human pathogen *Staphylococcus aureus* leads to abscesses in tissue, and in certain instances, it may cause mortality [1]. Methicillin-resistant *Staphylococcus aureus* (MRSA) is found in community and hospital settings, and it is resistant to several existing antibiotics, prompting an urgent need for alternative drugs to combat antibiotic-resistant pathogens [2]. Presently, vancomycin is widely in use to treat MRSA infections [3], but antimicrobial peptides (AMPs)—which are comprised of short amino acid sequences and serve as a first line of defense agent against invading pathogens in many species—have been suggested as potential highly efficacious alternatives for treating MRSA [4,5,6]. A synthetic AMP identified from the marine organism *Epinephelus coioides*, epinecidin-1 (Epi-1), exhibits antimicrobial and immunomodulatory functions against a range of pathogens [7]. The *Epinecidin-1* gene encodes a 67-amino acid pre-propeptide. A shorter 21-aa sequence from the C-terminal domain of epinecidin-1 propeptide (amino acids number 22–42) accounts for most of the antimicrobial and anti-tumor activity; for most studies, this propeptide is synthesized in sufficient amounts for experimentation [8,9,10]. Staphylococcal infections in the skeleton, lungs, and endocardium leads to the development of heart valve infection, bone inflammation, and abscess formation in lungs (pyemic lesions), which complicate sepsis [11]. MRSA is a major cause of sepsis-inducing nosocomial wound infections [12]. Sepsis is a major cause of morbidity and mortality in humans, and the rate of sepsis-related death has increased year by year [11]. 

In response to the induction of sepsis by pathogens, creatinine (CRE), blood urea-nitrogen (BUN), and uric-acid (UA) are elevated, indicating kidney injury. Thus, BUN, CRE, and UA levels are often monitored to assess pathogen infection-mediated abnormalities in the kidney, and drugs that control the levels of these molecules are considered effective protectants of kidney function [13]. In hepatocytes, organelle membrane damage causes swelling, hepatocyte necrosis, and the release of cytosolic glutamic oxaloacetic transaminase (GOT) and glutamic pyruvic transaminase (GPT) enzymes, which precede mortality from sepsis [14]. Hence, methods to attenuate BUN, CRE, UA, GPT, and GOT increases in blood following infection may prevent mortality as well. Intravenous *S. aureus* infection results in abscess formation in kidney, and can be tracked by bacterial counts in blood and visceral organs, such as the heart, liver, kidney, and lung [1]. The efficiency of Epi-1 in protecting against MRSA infection in mice models has been recently reported [15]. However, small animal models, such as mice or rats, cannot faithfully reproduce or mimic many aspects of human pulmonary system pathophysiology, and few reports have employed large animal models, such as swine or canine models, for MRSA infection studies [16]. 

In the present study, we investigated the efficacy of Epi-1 in a pyemia pig model, which is physiologically similar to MRSA infection in human beings. The injection of Epi-1 did not cause any observable side effects, and the peptide was stable in blood up to 4 h post-injection. When Epi-1 was injected 30 min after MRSA infection, BUN, CRE, UA, GPT, and GOT levels were controlled. Furthermore, Epi-1 provided complete protection against mortality when injected at a dose of 2.5 mg/kg. MRSA-mediated induction of the sepsis-associated molecule C-reactive protein (CRP), the proinflammatory cytokines IL-6, IL-1β, and TNF-α were also attenuated by Epi-1 treatment. Finally, Epi-1 also enhanced clearance of the MRSA pathogen from the blood, heart, liver, lungs, and kidney.

## 2. Results 

### 2.1. Epi-1 Inhibits MRSA

**Figure 1:**
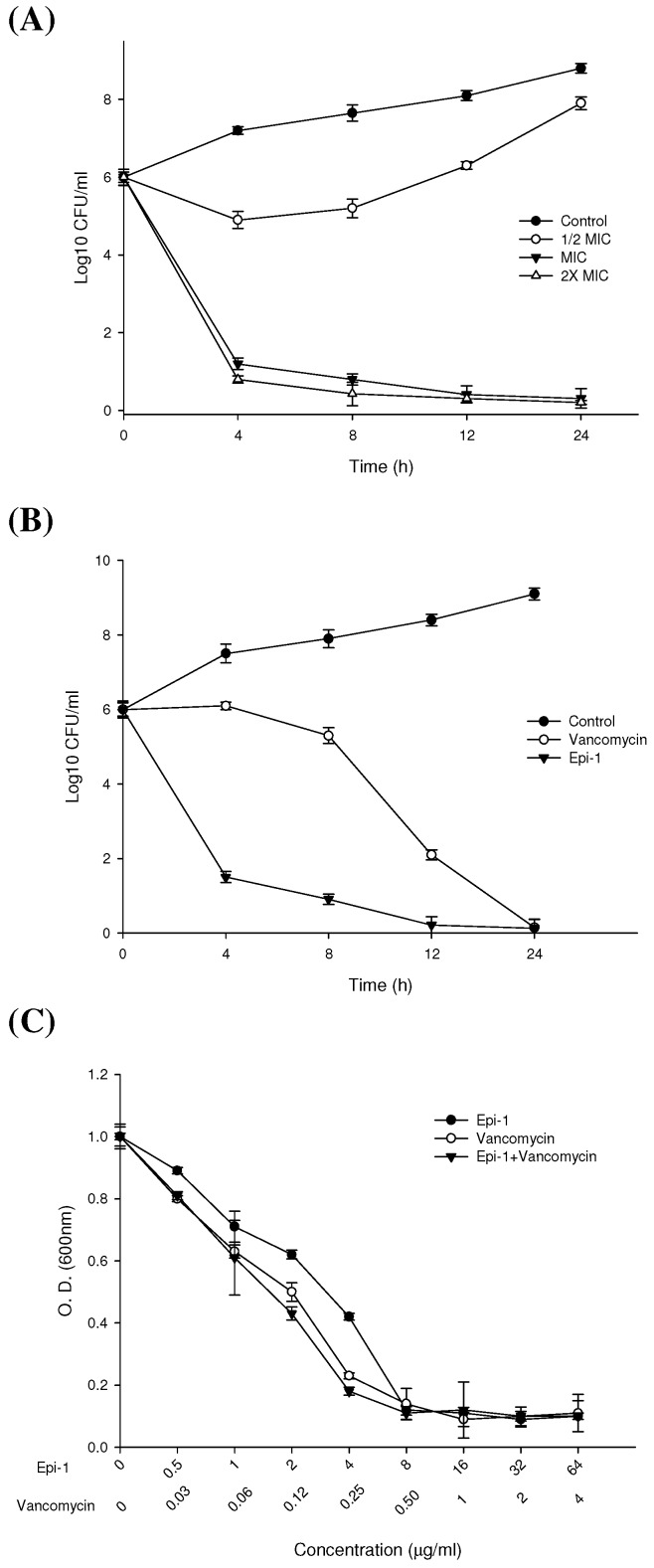
Antibacterial function of epinecidin-1 (Epi-1) toward MRSA is dependent on concentration and incubation time. (**A**) Dose and time-dependent bactericidal curves of 1/2, 1, and 2 × MIC concentrations of Epi-1 incubated with MRSA. MRSA alone was used as control. Samples were collected at 4, 8, 12, and 24 h post-exposure, and colony counts were determined. Each curve point represents the mean ± SEM. (**B**) Time-dependent killing effects of Epi-1 on MRSA. Approximately, 1 × 10^6^ cells were used to measure MIC of Epi-1 and the clinically used antibiotic vancomycin. The cultures were monitored for 24 h, and aliquots were taken at 4, 8, 12, and 24 h to determine the surviving CFU. The data represent means ± SEMs. (**C**) Combination effects of Epi-1 and an antibiotic were observed on MRSA. Epi-1 or Epi-1 with vancomycin. Each point represents the mean ± SEM of two independent experiments.

Given that cationic AMPs have broad-ranging antibacterial functions, these molecules have been suggested as alternatives to combat the increasingly common problem of MRSA infection. Concentrations of Epi-1 ranging between 0.5 and 64 µg/mL were initially studied in vitro, where Epi-1 exhibited strong inhibitory against MRSA. The effectiveness of this AMP against MRSA at a low dose is particularly important when considering it as a potential therapeutic. Relatively rapid clearance of pathogens by the AMP would ultimately reduce the overall exposure to MRSA. Figure 1A shows the dose and time-dependent antimicrobial function of Epi-1 against MRSA. Epi-1 antimicrobial activity was more rapid after treatment with higher concentrations. As such, 1 × MIC (minimum inhibitory concentration) and 2 × MIC killed 90% of pathogens at 4 h post-treatment, with a more gradual decrease of bacterial counts after 8 h (Figure 1A). At one-half the MIC, there was an initial drop in bacterial counts, but after 4–8 h post exposure the activity was lost. Thereafter, the bacteria regrew exponentially, similarly to the control samples. We further determined the temporal susceptibility profiles for MRSA (Figure 1B). After 4 h, Epi-1 reduced MRSA counts by > log 4, but vancomycin only showed 95% killing at 24 h. These findings suggest that the Epi-1 may be a strong antimicrobial agent for Gram-positive bacteria. Previous research results suggested that we should test the combination of Epi-1 and vancomycin [15]. The MIC values toward MRSA were measured, and Epi-1 inhibited bacterial growth to an OD600 less than 0.1 at doses from 6.25 µg/mL to 100 µg/mL. The ability of Epi-1 concentrations from 0.78 µg/mL to 3.125 µg/mL to inhibit growth was dose-dependent [10,15]. In order to keep drug costs to a minimum and to find the optimal experimental conditions, we tested the probable interaction effects of combinations of the two different antimicrobial agents. Epi-1, at concentrations ranging from 0.5 µg/mL to 64 µg/mL, was compared with vancomycin at 0.03–4 µg/mL. We also tested the antimicrobial activity of combined Epi-1 (0.5–64 µg/mL) with vancomycin (0.03–4 µg/mL) (Figure 1C), revealing that Epi-1 and vancomycin showed a combination effect on the MIC.

### 2.2. Administration of Epi-1 Does Not Cause Toxicity or Abnormal Clinical Signs in Pigs

**Figure 2:**
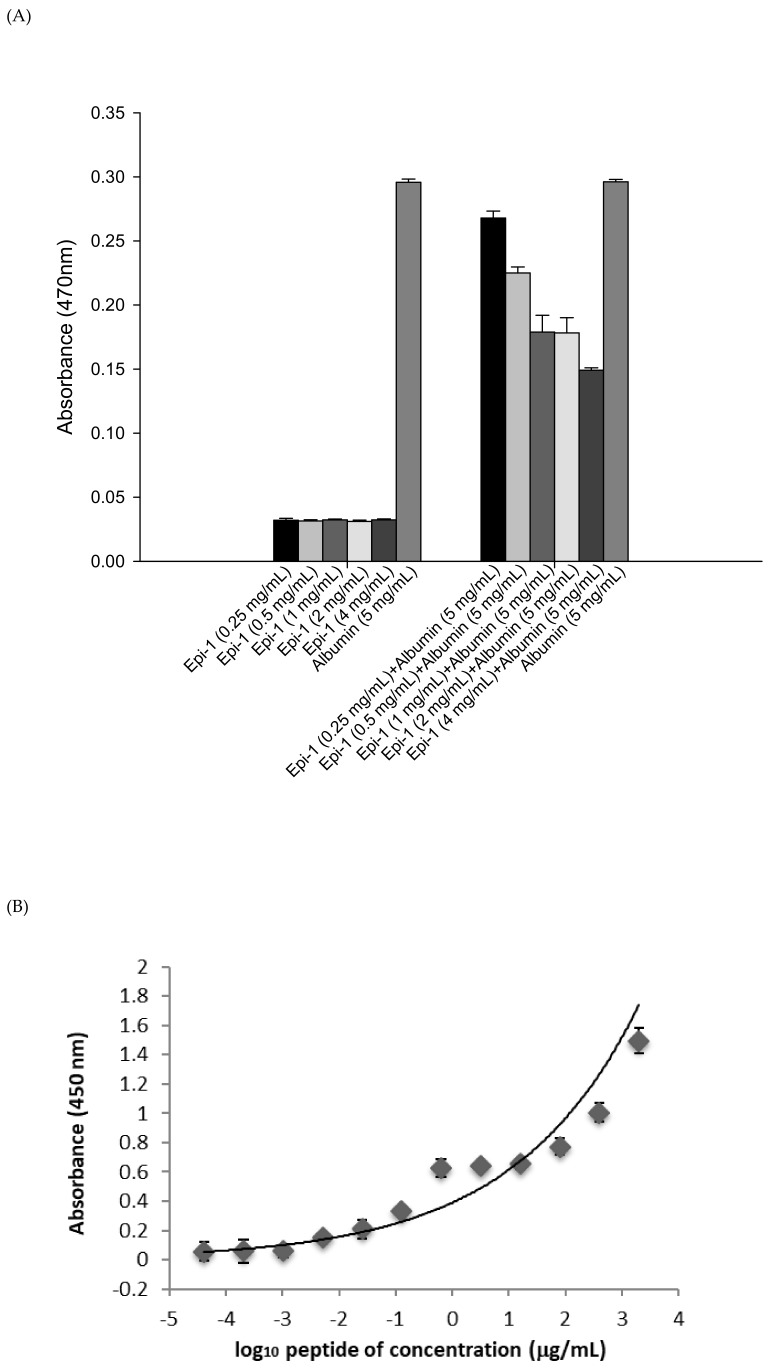

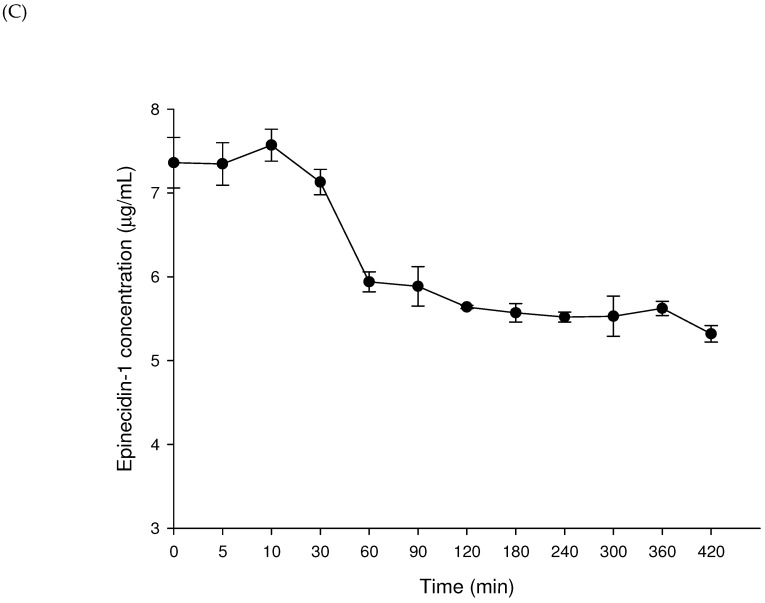
Epi-1 i.v. treatment did not alter serum biochemical parameters. Pigs were i.v. injected with 25 to 200 mg/kg body weight Epi-1 in PBS, and blood samples were collected after 24 h. (**A**) Co(II)–albumin binding test. Various doses of Epi-1 (0.25–4 mg/mL) were incubated with albumin (5 mg/mL). Then, cobalt chloride (1 g/L) and DTT (1.5 g/L) were added, and the absorbance of the mixtures was read at 470 nm. Albumin alone was used as a control. (**B**) Epi-1 binding to immobilized human albumin was detected with biotinylated Epi-1 followed by streptavidin-HRP in an albumin/Epi-1 sandwich ELISA. The data show means ± SEMs of three independent experiments. (**C**) Epi-1 in human serum was stable for up to 400 min in vitro. The data show means ± SEMs of three independent experiments.

Prior to testing the anti-MRSA activity of Epi-1 in pigs, we investigated whether intravenous (i.v.) injection of Epi-1 alone would harm the test animals. To assess the acute toxicity of the drug, acclimatized piglets were starved of food for 12 h and i.v. injected with 25, 50, 100, or 200 mg/kg Epi-1. The animals were observed every 2 h for morbidity or other clinical signs of toxicity for up to 24 h. Doses up to 100 mg/kg Epi-1 did not cause abnormal behavior, mortality, or activity, but a pig died soon after receiving a dose of 200 mg/kg. Serum UA, BUN, CRE, GOT, and GPT levels were within the normal range (Table 1) compared with normal pigs, confirming normal biochemical functions. In many instances, synthetic peptides lack conformational stability, which is a required quality for a successful drug. Therefore, the stability of potential peptide drugs in serum should be assessed to prevent the further development of unstable peptides in a drug development pipeline [17]. We determined Epi-1 stability in serum by liquid chromatography tandem mass spectroscopy (LC-MS/MS). The plasma-rich protein, human serum albumin (HSA), binds to a wide range of drugs, disrupting their delivery, decreasing efficacy and altering the drug pharmacokinetics and pharmacodynamics [18]. We used an albumin cobalt binding test to detect alterations in albumin configuration that reflect interactions between albumin and pharmaceutical formulations. In Figure 2A, albumin binding was noted at low concentrations of Epi-1 (0.25 mg/mL). Increased Epi-1 concentrations (1–4 mg/mL) further affected Co–DTT binding. This result supported the idea that the Epi-1 binds to albumin. To confirm the interaction, an albumin/Epi-1 sandwich assay was carried out. This sandwich assay showed binding of immobilized human albumin with biotinylated Epi-1 (Figure 2B). In addition, Epi-1 peptide stability in serum was assessed by LC-MS/MS (Figure 2C). The concentration of free Epi-1 was gradually decreased from 7.36 ± 0.30 to 5.94 ± 0.12 µg/mL in the initial 30 min. Thereafter, the concentration did not change (up to 7 h). Based on these results, we suspected that Epi-1 may bind to plasma protein (ex-albumin), resulting in half-life extension.

**Figure 3:**
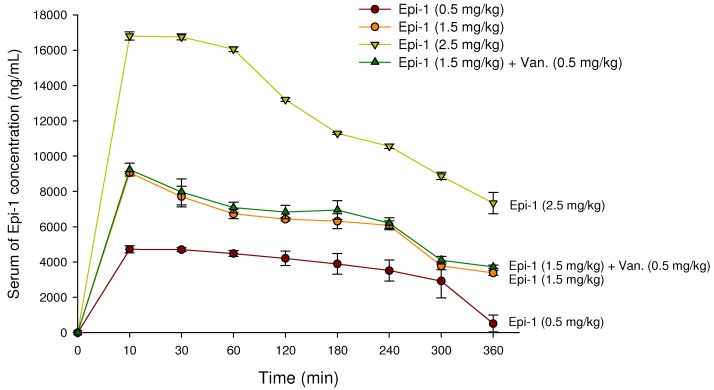
Pharmacokinetics of i.v. treated Epi-1 in pigs. Pigs were i.v. infused with 0.5, 1.5, or 2.5 mg/kg of Epi-1; 1.5 mg/kg Epi-1 plus 0.5 mg/kg vancomycin; or 0.5 mg/kg vancomycin in PBS. Blood samples were collected from the carotid artery after 0, 10, 30, and 60 min and every 60 min thereafter, until 360 min had elapsed. Serum was obtained from blood, and the concentration of Epi-1 was determined by liquid chromatography-mass spectrometry/mass spectrometry (LC-MS/MS). The data show means ± SEMs of two independent experiments.

For therapeutic drug monitoring, the plasma drug concentration is determined at certain intervals, allowing researchers to better define safety levels and effectiveness at specific doses [19]. We injected pigs i.v. with 0.5, 1.5, or 2.5 mg/kg Epi-1 either alone or with the addition of 0.5 mg/kg vancomycin in PBS buffer for pharmacokinetic experiments. Then, the stability of drug in serum was assessed using blood samples collected at 0, 10, 30, 60, 120, 180, 240, 300, and 360 min post-injection (Figure 3). The serum concentration decreased over time, from 16,808.06 ± 234.33 (Epi-1 2.5 mg/kg), 9061.33 ± 60.04 (Epi-1 1.5 mg/kg), 4720.00 ± 234.33 (Epi-1 0.5 mg/kg), 9240.00 ± 234.33 (Epi-1 1.5 mg/kg plus vancomycin 0.5 mg/kg) ng/mL at 10 min after injection to 8879.00 ± 205.23 (Epi-1 2.5 mg/kg), 3784.66 ± 219.31 (Epi-1 1.5 mg/kg), 2924.87 ± 957.48 (Epi-1 0.5 mg/kg), 4105.50 ± 210.00 (Epi-1 1.5 mg/kg plus vancomycin 0.5 mg/kg) ng/mL at 300 min. On the other hand, the serum concentration of vancomycin concentration was very stable (Supplementary Figure S1). From these results we found the bioavailability of Epi-1 was 60%–94% at 10 min injection (body weight of the pigs ranged from 10 to 13 kg, and blood weight was calculated as one-eighth the body weight; 1.25 to 1.62 kg). The biological half-life of Epi-1 in plasma was about 4 h. Hence, the peptide was administered every 3 h when we further studied the protective ability of Epi-1 against MRSA-mediated mortality in pigs.

### 2.3. Epi-1 Protects Pigs against MRSA-Mediated Mortality

**Figure 4:**
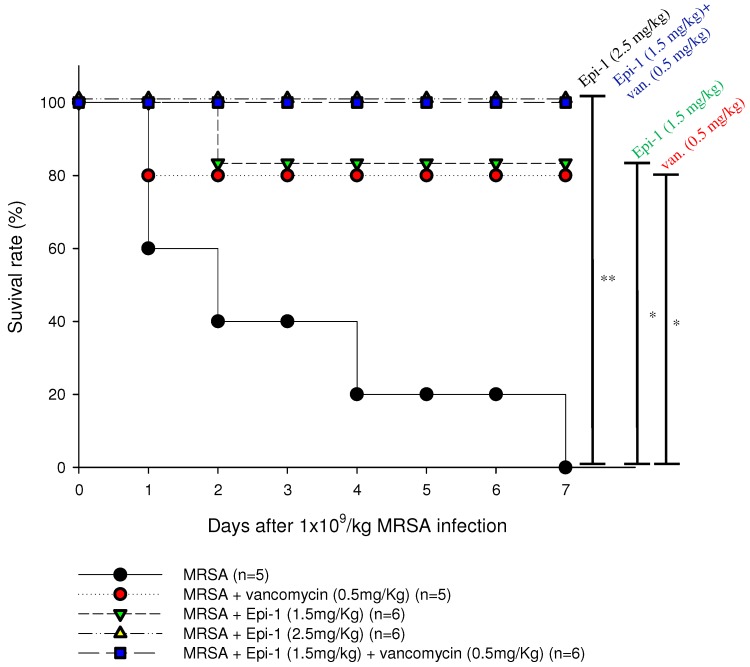
Epi-1 at 2.5 mg/kg body protects swine against MRSA infection. Pigs were i.v. injected with MRSA at 1 × 10^9^ CFU/kg, and 30 min post-infection, each pig was treated with an i.v. injection of 1.5 mg/kg Epi-1, 2.5 mg/kg Epi-1, 0.5 mg/kg vancomycin, or 1.5 mg/kg Epi-1 plus 0.5 mg/kg vancomycin. The survival rate was monitored for 7 days. * *p* < 0.05 and ** *p* < 0.01 as determined by a log-rank test.

In the pyemia pig, blood-poisoning model was established by injecting pigs i.v. with MRSA at 1 × 109 CFU/kg. A critical assessment of sepsis was performed by evaluating vital signs, including temperature and blood pressure (Supplementary Table S1). All pigs injected with MRSA alone died within seven days of infection due to blood poisoning and sepsis. To assess the curative potential of Epi-1 against MRSA infection, pigs were first infected with MRSA. Then, 30 min post-infection, the pigs received an injection of 1.5 mg/kg Epi-1, 2.5 mg/kg Epi-1, 0.5 mg/kg vancomycin, or 1.5 mg/kg Epi-1 plus 0.5 mg/kg vancomycin. The activity level of the pig, microbiological counts of MRSA, and sepsis-associated biochemical parameters (UA, BUN, CRE, GOT, and GPT) were assessed. All Epi-1- or vancomycin-treated pigs recovered and exhibited normal activity from day one onwards, but pigs infected with MRSA alone exhibited disease symptoms and reduced activity (Supplementary Video). While all pigs that received MRSA alone died within seven days of infection (Figure 4), the MRSA-infected pigs receiving Epi-1 or 1.5 mg/kg Epi-1 plus 0.5 mg/kg vancomycin exhibited 100% protection against MRSA infection (Figure 4). Infected pigs that received treatments of vancomycin alone exhibited 80% survival against MRSA infection (Figure 4). These results showed that that Epi-1 conferred complete protection against MRSA and was better than the existing antibiotic drug vancomycin.

### 2.4. Epi-1 Regulates Serum C-Reactive Protein and Plasma IL6, IL1β, and TNFα, While Mitigating Elevations of UA, BUN, CRE, GOT, and GPT in MRSA-Infected Pigs

**Figure 5:**
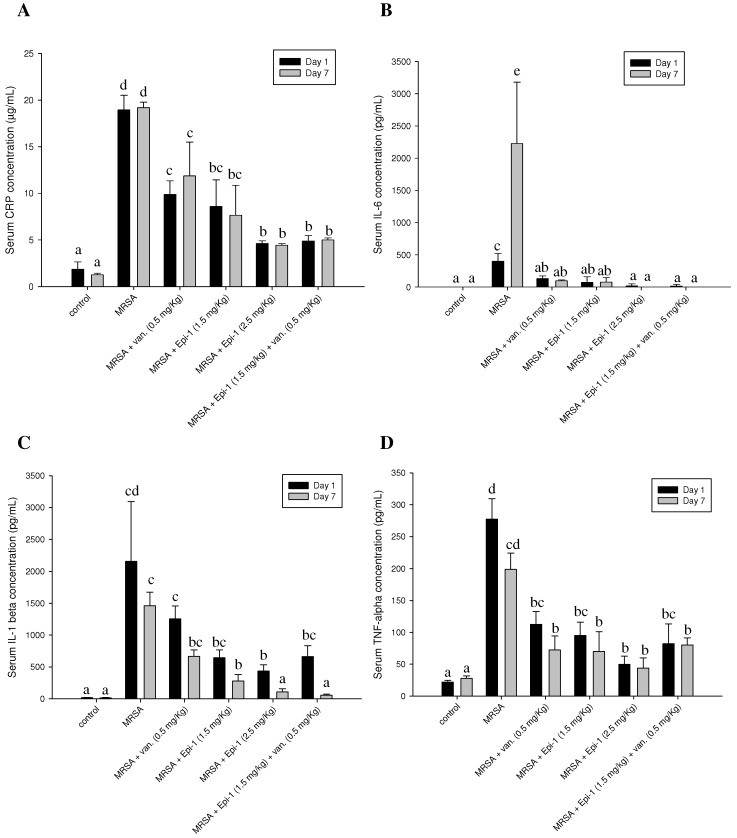
Epi-1 inhibits the MRSA-mediated induction of sepsis markers CRP, IL6, IL1β, and TNFα. Pigs were i.v. injected with MRSA at 1 × 10^9^ CFU/kg, and 30 min post-infection, the pigs were treated with i.v. injection of 1.5 mg/kg Epi-1, 2.5 mg/kg Epi-1, 0.5 mg/kg vancomycin, or 1.5 mg/kg Epi-1 plus 0.5 mg/kg vancomycin. Serum concentrations of CRP, IL6, IL1β, and TNFα were determined at various time points after infection with MRSA. Data with different letters indicate significant differences (*p* < 0.05) between treatments. (**A**) CRP; (**B**) IL6; (**C**) IL1β; (**D**) TNFα.

During inflammation and sepsis, plasma C-reactive protein (CRP), IL-6, IL-1β, and TNF-α levels in the circulatory system are increased. Plasma was obtained from heparin-stabilized blood, and ELISA was used to detect CRP, IL-6, IL-1β, and TNF-α proinflammatory cytokines. Pyemia pigs exhibited elevated CRP, IL-6, IL-1β, and TNF-α (Figure 5). In pigs that received a curative dose of Epi-1, the elevated levels of CRP, IL-6, IL-1β, and TNF-α were reduced (Figure 5). Furthermore, UA, BUN, CRE, GOT, and GPT levels were measured and compared between pigs treated with MRSA alone, MRSA with Epi-1, or MRSA with vancomycin (Table 2). The levels of UA, BUN, CRE, GOT, and GPT were elevated in MRSA-infected pigs, indicating the devastating effects of MRSA on the function of various organs, such as kidney and liver, and blood (Table 2).

### 2.5. Epi-1 Clears MRSA from Infected Pigs

**Figure 6:**
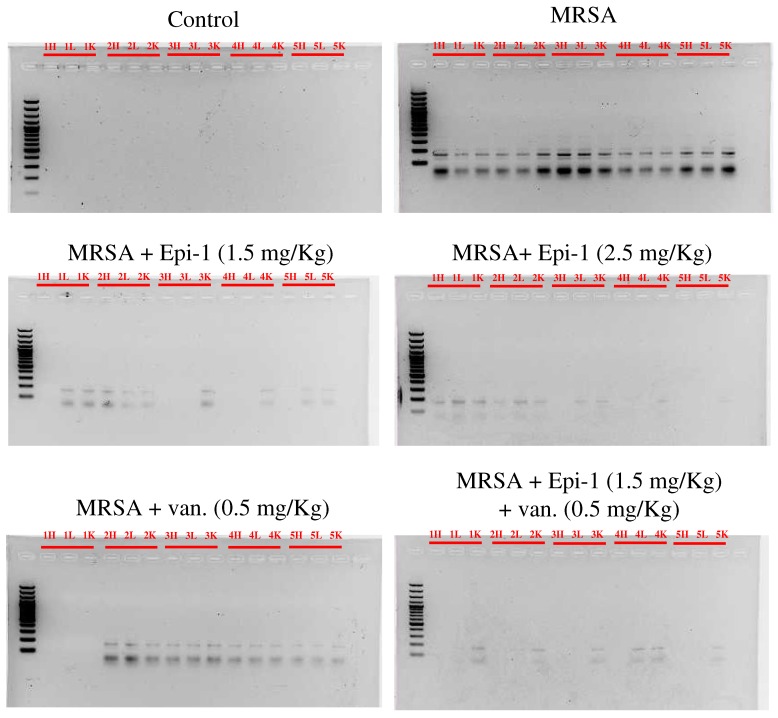
Epi-1 decreases MRSA count in infected pigs. Pigs were i.v. injected with MRSA at 1 × 10^9^ CFU/kg, and 30 min post-infection, the pigs were treated with i.v. injection of 1.5 mg/kg Epi-1, 2.5 mg/kg Epi-1, 0.5 mg/kg vancomycin, or 1.5 mg/kg Epi-1 plus 0.5 mg/kg vancomycin. Multiplex PCR amplification was performed using *Staphylococcus aureus* (SA)-specific primers for etB (226 bp), mecA (163 bp), femA (132 bp), and etA (93 bp) and visualized by agarose gel electrophoresis. Numbers 1 to 5 indicate the group of the pig. Heart (H), MRSA-specific bands were not detectable or decreased in intensity in Epi-1 and Epi-1 plus antibiotic-treated pigs. H, L, and K indicate heart, lung, and kidney, respectively.

**Figure 7:**
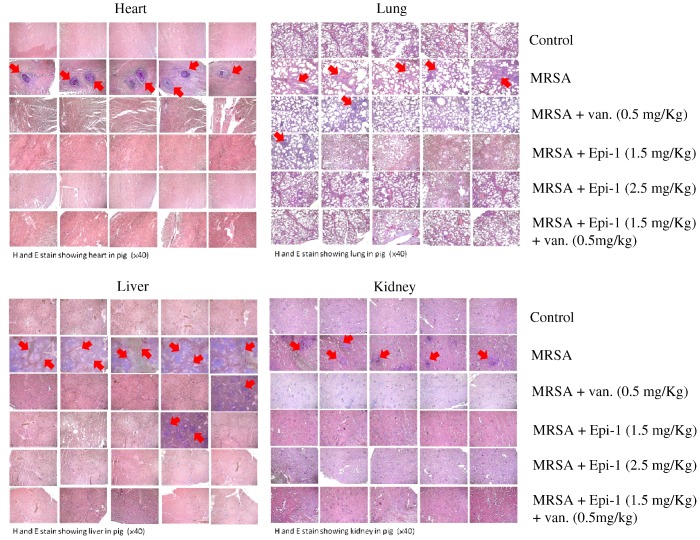
Epi-1 improves the pathology of MRSA infected pigs. Pigs were i.v. injected with MRSA at 1 × 10^9^ CFU/kg, and at 30 min post-infection, the pigs were treated with i.v. injection of 1.5 mg/kg Epi-1, 2.5 mg/kg Epi-1, 0.5 mg/kg vancomycin, or 1.5 mg/kg Epi-1 plus 0.5 mg/kg vancomycin. Heart, lung, liver, and kidney sections were fixed for 24 h in 4% formaldehyde in PBS and subjected to H&E staining. After staining, the samples were observed under a microscope. Red arrows indicate tissue lesions and necrosis. Data are representative of five pigs in each group.

Multiplex PCR specific to SA endotoxin is an efficient method with which to detect the presence of pathogens in a host organism [20,21]. To measure endotoxin neutralization efficiency, genomic DNA was obtained from the hearts, livers, and kidneys of pigs, and multiplex PCR was performed with primers specific to *etB* (226 bp), *mecA* (163 bp), *femA* (132bp), and *etA* (93 bp) genes. The sequences corresponding to *etA* (93 bp) and *mecA* (163 bp) were amplified in hearts, livers, and kidneys of pigs infected with MRSA alone, confirming the presence of the pathogen (Figure 6). The expression levels of *etA* (93 bp) and *mecA* (163 bp) were significantly reduced in the Epi-1 or Epi-1-plus-vancomycin-treated pigs (Figure 6). This result demonstrates the ability of Epi-1 to reduce the level of endotoxins. To further confirm that the suppression of etA (93 bp) and mecA (163 bp) was due to the direct antibacterial function of Epi-1, blood samples were cultured in mannitol salt agar (MSA) medium with oxacillin 2 µg/mL. MRSA counts were made from samples collected 10 min before infection and 1, 2, 3, 4, 5, 6, and 7 days post-infection (Table 3). In pigs that received Epi-1 at 2.5 mg/kg, counts were not detectable from day 2 onward (Table 3). In pigs treated with Epi-1 at 1.5 mg/kg or 1.5 mg/kg Epi-1 plus vancomycin, counts were not detectable from day 4 day onward (Table 3). Bacterial colonization in other organs was examined by culturing tissue homogenates of the heart, lung, liver, and kidney (Table 4). Bacterial counts were not detected in pigs that received 2.5 mg/kg of Epi-1 or 1.5 mg/kg Epi-1 plus 0.5 mg/kg vancomycin, but all other treatment groups, including 0.5 mg/kg vancomycin, showed bacterial counts in all organs (Table 4). Hematoxylin and eosin staining may allow for the visualization of a range of pathogens, such as bacteria, fungi, protozoa, and viruses. Therefore, we harvested hearts, lungs, livers, and kidneys of infected pigs on day 7 and performed hematoxylin and eosin staining to evaluate tissue pathology. Control and various dosages of Epi-1 treatment plus the vancomycin group showed normal tissue architecture of the heart, lung, liver, and kidney. Pigs infected with MRSA had necrotic lesions in the heart, lung, liver, and kidney, which are commonly damaged organs (Figure 7 and Table 5).

## 3. Discussion

*S. aureus* persistently colonizes the nostrils and causes skin, soft-tissue, and blood infections. Approximately one-fifth of the human population is at risk of infection [22]. Surgical-site infection is a common healthcare-associated source of *S. aureus* infection, which often causes morbidity [23]. The emergence of multi-drug resistant *S. aureus* necessitates the development of novel therapies to cure the multi-drug resistant pathogens [24]. AMPs are promising alternative therapeutics to combat such multi-drug resistant strains [15]. MRSA is increasingly commonly encountered in clinical environments and communities [25,26]. Previous reports demonstrated that an AMP from a marine organism, Epi-1, provides complete protection against MRSA infection in mice models [11]. Large animal models, such as swine, may mimic or reproduce many aspects of human pulmonary pathophysiology, but investigations into the therapeutic potential of AMPs in such models are limited [27]. Since studies of larger animal models may be more relevant to human pathology and drug efficiency [27,28], we investigated the efficacy of Epi-1 in a MRSA-induced sepsis-associated swine model [29]. This pyemia pig model was utilized based on its ability to mimic human pathophysiology [18].

Establishing safe, appropriate dosages and correct usage are important goals for preclinical trials [30]. Epi-1 showed effective in vitro bactericidal activity (Figure 1A–C), and intravenous single-dose injection of 25, 50, or 100 mg/kg of Epi-1 did not cause any apparent side effects. The biochemical parameters of BUN, CRE, UA, GPT, and GOT were not elevated in pigs injected with 25, 50, or 100 of Epi-1 (Figure 2A). Thus, we concluded that administration of Epi-1 did not cause adverse effects to the blood, lungs, kidney, heart, or liver (Figure 2B,C). We further detected Epi-1 in serum up to 240 min post-injection (Figure 2D and Figure 3), demonstrating that this peptide is stable and safe for clinical trials. The survival study revealed that Epi-1 at 2.5 mg/kg provided complete protection against MRSA infection in pigs, whereas the clinically approved antibiotic, vancomycin, only protected 80% of pigs from mortality (Figure 4). These data suggest that Epi-1 was more effective than vancomycin at protecting MRSA-infected pigs. The plasma markers of inflammation, CRP and IL6, are known to be elevated during inflammation-mediated sepsis and lead to mortality [28]. Hence, inflammatory cytokines must be kept under control [28]. The MRSA-mediated inductions of CRP, IL6, IL1β, and TNFα were efficiently controlled by Epi-1, confirming the suppression of inflammation by Epi-1 (Figure 5). Multiplex PCR with *S. aureus*-specific primers and hematoxylin and eosin staining of tissue samples demonstrated that Epi-1 efficiently reduced bacterial counts in MRSA-infected pigs (Figure 6; Figure 7 and Table 4). 

Altogether, sepsis is a leading cause of illness and mortality in hospital patients around the globe each year. Currently available treatments, such as systemic antibiotics, carry the unavoidable risk of promoting the emergence of antibiotic-resistant pathogen strains. The identification of novel alternative therapeutic agents with anti-sepsis and antimicrobial functions is necessary in order to decrease uncontrolled inflammatory responses. Our previous studies have clearly demonstrated that the AMP Epi-1, from the marine organism *Epinephelus coioides*, efficiently protects against MRSA in vitro and in vivo. However, most previous experiments were performed with small animal models, such as mice or rats, which cannot reproduce many aspects of human pulmonary pathophysiology. This study shows that the Epi-1 is stable up to 4 h post i.v. injection in serum without causing any apparent toxic effects, and it efficiently protects against MRSA infection in a pyemia pig model. Thus, Epi-1 may be a promising candidate as an alternative drug to cure MRSA infection. Overall, Epi-1 appears to be a valuable compound for the development of new antimicrobial drugs and may be useful as a therapeutic for sepsis patients.

## 4. Material and Methods 

### 4.1. Experimental Pigs, Pathogen, and Peptide

Yorkshire breed healthy pigs were purchased at 6 weeks of age and 10 to 13 kg bodyweight from the pig herd at Pingtung, Taiwan. The pigs were acclimated to the animal facility, thoroughly examined, and confirmed to be free from clinical signs and disease prior to experimentation [11]. Animal housing, handling, and in vivo experimental procedures were performed in strict accordance with instructions and guidelines of National Pingtung University of Science and Technology (NPUST) and approval from the Animal Care and Use Committee of NPUST. The experimental pigs were given intramuscular injections of 2 mg/kg xylazine (Balanzine, Health-tech Pharma, Taipei, Taiwan) and 20 mg/kg ketamine (Imalgene 1000, Merial-Taiwan, Taipei, Taiwan) to anesthetize the animals. Injections, insertion of catheters, blood sample collection, and surgical procedures were performed under aseptic conditions with 70% ethanol disinfectant. Synthetic Epi-1 with a GFIFHIIKGLFHAGKMIHGLV-NH2 amino acid sequence and produced by an FMOC system was purchased from GL Biochemistry (Shangai, China). Lyophilized Epi-1 was suspended in phosphate-buffered saline (PBS; pH 7.4) prior to experiments [15]. The MRSA strain was obtained from a stool isolate obtained from Taipei City Hospital (Heping Fuyou branch, Taipei, Taiwan), and its resistance to the antibiotics ampicillin, methicillin, oxacillin, and ciprofloxacin was confirmed. MRSA in vitro culture and quantification followed previous publications [15,31,32].

### 4.2. In Vitro MRSA Growth Inhibition Assay 

Antibacterial properties were assessed according to the MIC values. A micro-dilution assay was performed with 200 µL volume in 96-well sterile plates. MRSA with 0.08–0.1 OD was prepared, and was followed by a 1000-fold dilution using TSB broth. Diluted MRSA cultures were aliquoted at 180 µL/well. Epi-1 was prepared (0.5–64 µg/mL) immediately prior to adding 20 µL to each well; the control was PBS. The experimental plate was incubated at 37 °C for 16 h. Growth performance was determined by measuring the absorbance at 600 nm in an ELISA plate reader. The MIC was determined as the lowest Epi-1 concentration that showed minimal absorbance after 24 h of incubation [33].

### 4.3. Time-Kill Kinetics

MRSA cultured for 16 h was diluted to 0.1 OD, and the culture inoculums were mixed with Epi-1 at 0.5, 1 and 2 times the MIC, followed by culturing at 37 °C. At 4, 8, 12, and 24 h post-treatment, the number of viable bacteria (CFU) was determined for the time-kill experiment [33].

### 4.4. Comparative Efficacy Dose of Epi-1 and Antibiotics

The antimicrobial function of Epi-1 and vancomycin against MRSA was assessed by the dose-dependent decrease in OD. MRSA (0.1 OD) was mixed with 0.5 to 64 µg of Epi-1, and 0.03 to 4 µg of vancomycin. The mix was then incubated for 16 h at 37 °C, and followed by measurement of light absorption at OD600.

### 4.5. Acute Toxicity Studies

Acute intravenous (i.v.) toxicity was analyzed as described previously [34]. Acute i.v. toxicity was assessed after administration of 25, 50, 100, and 200 mg/kg body weight of Epi-1 in pigs; 1 to 5 animals per group. After injection, the animals were kept under visual observation up to 1 h, and the rate of mortality and clinical symptoms were recorded every 2 h for a total of 24 h. For blood analysis, 5 mL of heparin-stabilized blood was taken and stored at 4 °C. Serum was separated from the blood sample using a serum separator gel tube (catalogue number 367955, Becton-Dickinson, Franklin Park, NJ, USA), according to the vendor’s instructions. Serum GOT, GPT, UA, BUN, and CRT activity levels were determined using an automated analyzer (FUJI DRI-CHEM 4000i, Fujifilm, Tokyo, Japan) according to the manufacturers’ instructions. Standard controls were analyzed before each determination.

### 4.6. Spectrophotometric Co(II) Albumin Binding Assay

The concentration of albumin was indirectly determined by adding a specific amount of Co(II) to the test item, followed by measuring the free Co(II) by a colorimetric assay with dithiothreitol (DTT) (35). A negative relationship exists between the amount of albumin-bound Co(II), and the intensity of color in the assay. The reaction was performed at ambient temperature in a 1.5 mL centrifuge tube. To perform the assay, 100 µL of albumin (50 g/L) was mixed with 0.25, 0.5, 1, 2, or 4 mg/mL Epi-1 in 50 µL of 1.0 g/L cobalt chloride solution, and that was incubated for 10 min. Next, 50 µL of 1.5 g/L DTT solution was added, and the solution was incubated for 2 min. Finally, 1.0 mL of 9 g/L concentrated NaCl solution was added, and the absorbance at 470 nm was read on a SpectraMax^®^ i3 (San Jose, CA, USA). DTT was excluded in the blank. All the chemicals used in this assay were purchased from Sigma-Aldrich (St. Louis, MO, USA).

### 4.7. Albumin/Epi-1 Sandwich Assay

Microtiter plates were coated with human serum albumin at 0.4 µg/well, and blocked with 200 μL of 5% non-fat dry milk powder in PBS containing 0.05% Tween 20 at 37 °C for 2 h. After blocking, the wells were washed with washing buffer that consisted of 0.05% Tween 20 in PBS. Next, 50 μL of serially diluted Epi-1 solution in PBS was added to each well and incubated 1 h at room temperature. The wells were then washed with washing buffer and incubated with streptavidin-HRP conjugate for 1 h. Then the mix was aspirated, washed with washing buffer, and 50 μL of BD OptEIATM ELISA detection agent (catalogue number 555214, BD biosciences, San Jose, CA, USA) was added. Finally, the aliquot was incubated for 30 min at room temperature in the dark, and the light absorbance at 450 nm (OD450) was read with an ELISA plate reader to measure peroxidase activity.

### 4.8. In Vitro Peptide Stability in Serum

Human serum (catalogue number BP2657100, Fisher Scientific, Millersburg, PA, USA) was suspended in RPMI medium up to 25%, and 0.990 mL was transferred to a 1.5 mL micro centrifuge tube. The serum-containing tube was incubated at 37 °C for 15 min, and 10 mg/mL Epi-1 was added to obtain a final peptide concentration of 100 μg/mL. At constant time intervals 100 μL of the experimental mix was taken out, and serum protein was precipitated in two volumes of 95% ethanol solution. The cloudy precipitate was cooled at 4 °C for 15 min, and then the serum was precipitated and pelleted by centrifugation at 18,000 × *g* for 2 min. The peptide stability in the supernatant was then analyzed by a liquid chromatography tandem mass spectrometer (LC-MS/MS) (Mission Biotech, Taipei, Taiwan).

### 4.9. Pharmacokinetics

An i.v. catheter was inserted into the femoral vein of each pig and infused with 0.5, 1.5 or 2.5 mg/kg of Epi-1; 1.5 mg/kg Epi-1 plus 0.5 mg/kg vancomycin; or 0.5 mg/kg vancomycin in PBS for pharmacokinetic analysis. Approximately 3 mL of blood was taken from the carotid artery at 0, 10, 20, 30, and 60 minutes and at 60 minutes intervals thereafter until 600 minutes had passed. Serum was obtained from the blood sample, and the Epi-1 level was measured by LC-MS-MS at Mission Biotech, Taiwan.

### 4.10. Induction of Sepsis

Administration of bacteria or mock treatment followed by flushing with 5 mL of sterile isotonic saline was performed by i.v. through a 22G catheter inserted in the right femoral vein. The catheter was removed after administration. Another 22G catheter was then inserted into the left femoral vein and secured with stitches. The catheter was flushed with 10 mL sterile isotonic saline solution, and then with 2 mL of 100 I.U./mL sterile heparin solution (catalogue number H3149, Sigma Chemical Co., St. Louis, MO, USA) for blood sampling. During blood sampling, the initial 5 mL of blood was discarded to avoid the heparin carryover. Five pigs were inoculated with the MRSA strain (1 × 109 CFU/kg) only. In addition, five pigs were injected with vancomycin (0.5 mg/kg) with sterile isotonic saline; six pigs were injected with Epi-1 (1.5 mg/kg) with sterile isotonic saline; six pigs were injected with Epi-1 (2.5 mg/kg) with sterile isotonic saline; and six pigs were injected with Epi-1 (1.5 mg/kg) plus vancomycin (0.5 mg/kg) with sterile isotonic saline 30 after MRSA strain infection. Samples were subsequently collected for histopathology, microbial studies, and bacterial count.

### 4.11. Detection of Bacterial Counts in the Blood, Heart, Lung, Liver, and Kidney

Blood samples were diluted to a 1 mL final volume with sterile isotonic saline solution and added to a sterile petri dish, where the samples were mixed with 25 mL melted mannitol salt agar (MSA) (catalogue number 7143, NEOGEN, Lansing, MI, USA). When the mixture reached a temperature of 45–50 °C, oxacillin (catalogue number 28221, Sigma) was added up to 2 µg/mL. The mixture was then cultured in a 37 °C incubator, and the bacterial count was calculated after 48 h. To detect bacterial counts in organs, 1 g of tissue from the heart (valve), lung (left diaphragmatic lobe), liver (left lateral lobe), and kidney (left) was sampled upon euthanasia or death from infection. The samples were collected and stored for 12 h at 4 °C. Tissues were then removed aseptically, cut into small pieces with a sterile blade, weighed to 1 g, and added to 9 mL of sterile isotonic solution for homogenization. A 10 μL aliquot of the dilution was used to inoculate the surface of melted MSA with 2 µg/mL oxacillin and cultured in a 37 °C incubator for 48 h. After incubation, microbial counts per gram tissue were calculated.

### 4.12. Quantification of Serum C-Reactive Protein and Plasma IL6, IL1β, and TNFα

Blood samples stabilized with heparin were immediately centrifuged in an endotoxin-free vial, and plasma was obtained. The plasma samples were initially stored in a 4 °C refrigerator for 1 h, and then transferred to a −80 °C freezer until use. Serum CRP content was measured with an ELISA kit (catalogue number KA1920, Abnova, Walnut, CA, USA). Plasma IL6 content was determined with an IL6-ELISA kit (catalogue number ab100755, Abcam, Cambridge, MA, USA). IL1β content in the plasma was determined with an IL1β-ELISA kit (catalogue number ab100754, Abcam). TNFα content in the plasma was measured with a TNFα-ELISA (catalogue number ab100756, Abcam), according to the supplier’s instructions.

### 4.13. Multiplex PCR Detection of the MRSA Strain

Genomic DNA was isolated from the hearts, livers, and kidneys of animals in each experimental group, using a Novel genomic DNA mini kit (catalogue number NG-S100, Novelgene, Taipei, Taiwan) by the manufacturer’s recommendations. *S. aureus*-specific oligonucleotide primer sequences were taken from previous reports (Supplementary Table S2) [21,35,36]. A GeneAmp kit (Perkin-Elmer, Norwalk, Conn., Waltham, MA, USA) was used to perform PCR amplification of primer regions in the genomic DNA, following the supplier’s protocol. The multiplex PCR mix contained dNTPs, 10 × reaction buffer (100 mM Tris-HCl pH 8.3, 500 mM KCl); 1.5 mM MgCl2; SA-specific *etB*, *etA*, *mecA*, and *femA* primers at 20 pmol each; Taq DNA polymerase; and approximately 100 ng of DNA template. The final volume was made up to 50 μL with nuclease free sterile distilled water. DNA amplification was performed in a thermocycler (catalogue number 4375305, Thermo Scientific, Waltham, MA, USA) with the initial denaturation at 94 °C for 5 min, 35 cycles of amplification at 94 °C for 2 min, 57 °C for 2 min, and 72 °C for 1 min. At the end of 35 cycles a final extension at 72 °C for 7 min (followed by holding at 4 °C) was performed.

### 4.14. Histopathology Staining

Tissue samples from the heart valve, left diaphragmatic lobe of the lung, left lateral lobe of the liver, and left kidney were collected and fixed in PBS buffer containing 4% formaldehyde for 24 h. Fixed samples were immersed in different concentrations of xylene and ethanol before embedding in paraffin. The processed samples were sectioned at 8–10 µm using a microtome. The sections were stained for histological analysis with hematoxylin and eosin (H&E) after rehydration.

### 4.15. Statistical Analyses

Significant differences among treatments were identified with univariate ANOVA performed on SPSS statistical software 18.0 (SPSS Inc., Chicago, IL, USA). Log-rank test was used to analyze survival rate. The error bars show standard deviations or standard errors of the means (SEMs) as indicated. Significant differences at *p* < 0.05 or *p* < 0.01 are indicated. Significantly different values are indicated by different letters.

## Figures and Tables

**Table 1:** Serum uric acid (UA), blood urea nitrogen (BUN), creatinine (CRE), serum glutamic oxaloacetic transaminase (GOT), and serum glutamic pyruvic transaminase (GPT) levels were measured.

Dose (mg/kg)	Number of Pig	UA (mg/dl)	BUN (mg/dl)	CRE (mg/dl)	GOT (U/l)	GPT (U/l)
25	3	0.27 ^A^ ± 0.06	13.33 ^A^ ± 0.21	2.27 ^AB^ ± 0.21	59.33 ^A^ ± 3.79	52.00 ^A^ ± 15.72
50	3	0.23 ^A^ ± 0.06	14.63 ^A^ ± 2.95	1.90 ^A^ ± 0.20	59.67 ^A^ ± 8.08	45.33 ^A^ ± 4.04
100	3	0.27 ^A^ ± 0.06	14.67 ^A^ ± 2.89	1.87 ^A^ ± 0.15	61.67 ^A^ ± 10.50	58.00 ^A^ ± 14.93
200	1	Died	Died	Died	Died	Died
Control	5	0.26 ^A^ ± 0.05	13.18 ^A^ ± 1.47	1.92 ^A^ ± 0.41	56.20 ^A^ ± 14.11	49.00 ^A^ ± 13.04

Different letters indicate a significant difference between two groups. Died: pig died after injected dose 200 mg/kg in one hour.

**Table 2:** Blood UA, BUN, CRE, GOT, and GPT levels in MRSA-infected pigs at 30 min after i.v. injection with different combinations of Epi-1 and vancomycin. The samples were collected seven days post-infection either from euthanized pigs or immediately after the deaths of the pigs.

Group/Number	Inoculation	Parameters	Blood Sampling Time-Points (Time after Infection)			
			−30 min before	1 day	3 days	7 days
1/5	MRSA	UA (mg/dL)	0.31 ^A^ ± 0.01	0.36 ^AB^ ± 0.12	0.42 ^B^ ± 0.1	0.46 ^B^ ± 0.08
		BUN (mg/dL)	8.04 ^A^ ± 3.43	21.74 ^B^ ± 3.12	35 ^D^ ± 9.23	32.36 ^C^ ± 7.58
		CRE (mg/dL)	1.48 ^A^±0.17	2.12 ^B^±0.12	2.22 ^C^ ± 0.04	2.42 ^D^ ± 0.19
		GOT (U/L)	47.21 ^A^ ± 9.03	73 ^B^ ± 12.82	115 ^C^ ± 32.64	162 ^D^ ± 56.04
		GPT (U/L)	43.00 ^A^ ± 7.44	88.8 ^C^ ± 32.19	121.2 ^D^ ± 43.56	138.20 ^CD^ ± 65.09
2/5	MRSA + Vancomycin (0.5 mg/kg)	UA (mg/dL)	0.21 ^A^ ± 0.03	0.32 ^A^ ± 0.23	0.22 ^A^ ± 0.07	0.23 ^A^ ± 0.12
		BUN (mg/dL)	7 ^A^ ± 1.21	18.1 ^BC^ ± 5.46	15.2 ^B^ ± 0.61	13.11 ^B^ ± 1.32
		CRE (mg/dL)	1.3 ^A^ ± 0.07	2.4 ^B^ ± 0.37	2.12 ^AB^ ± 0.91	2.42 ^AB^ ± 1.12
		GOT (U/L)	37 ^A^ ± 10.32	78.22 ^B^ ± 21.65	42.41 ^A^ ± 6.21	52.32 ^B^ ± 11.24
		GPT (U/L)	40 ^A^ ± 8.11	87.67 ^B^ ± 38.22	54.01 ^AB^ ± 7.2	55.11 ^AB^ ± 4.02
3/6	MRSA + Epi-1 (1.5 mg/kg)	UA (mg/dL)	0.32 ^A^ ± 0.12	0.26 ^A^ ± 0.04	0.31 ^A^ ± 0.04	0.23 ^A^ ± 0.05
		BUN (mg/dL)	6.21 ^A^ ± 1.25	15.13 ^B^ ± 2.16	13.2 ^B^ ± 1.82	12.01 ^B^ ± 0.54
		CRE (mg/dL)	1.23 ^A^ ± 0.01	2.51 ^B^ ± 0.17	2.24 ^B^ ± 0.16	2.67 ^B^ ± 0.53
		GOT (U/L)	38.2 ^A^ ± 4.55	68.62 ^BC^ ± 16.21	38.2 ^A^ ± 14.23	59.05 ^B^ ± 1.35
		GPT (U/L)	47 ^A^ ± 9.11	67.36 ^B^ ± 15.33	67.1 ^B^ ± 9.34	75.32 ^C^ ± 10.14
4/6	MRSA + Epi-1 (2.5 mg/kg)	UA (mg/dL)	0.22 ^A^ ± 0.02	0.23 ^A^ ± 0.02	0.19 ^A^ ± 0.08	0.22 ^A^ ± 0.04
		BUN (mg/dL)	7.22 ^A^ ± 2.42	13.2 ^C^ ± 2.31	11.21 ^C^ ± 1.27	10 ^B^ ± 4.07
		CRE (mg/dL)	1.67 ^A^ ± 0.21	1.92 ^A^ ± 0.08	2.31 ^B^ ± 0.12	2.21 ^B^ ± 1.01
		GOT (U/L)	37.2 ^A^ ± 7.34	52.22 ^B^ ± 15.21	48.62 ^AB^ ± 21.13	72.33 ^C^ ± 30.73
		GPT (U/L)	48.2 ^A^ ± 9.23	60.33 ^B^ ± 21.01	71.04 ^C^ ± 17.61	51.33 ^AB^ ± 10.11
5/6	MRSA + Epi-1 (1.5 mg/kg) + vancomycin (0.5 mg/kg)	UA (mg/dL)	0.21 ^A^ ± 0.02	0.22 ^A^ ± 0.02	0.3 ^AB^ ± 0.13	0.28 ^A^ ± 0.04
		BUN (mg/dL)	7.32 ^A^ ± 2.13	16.12 ^B^ ± 1.24	14.22 ^B^ ± 1.67	10.56 ^AB^ ± 2.77
		CRE (mg/dL)	2.36 ^A^ ± 0.21	2.22 ^A^ ± 0.45	2.11 ^AB^ ± 0.82	1.41 ^B^ ± 0.16
		GOT (U/L)	37.5 ^A^ ± 4.34	51.26 ^B^ ± 10.16	52.31 ^B^ ± 10.52	61.16 ^C^ ± 17.11
		GPT (U/L)	47.2 ^A^ ± 1.59	60.03 ^C^ ± 12.44	56.34 ^B^ ± 5.21	50 ^AB^ ± 14.22

Values are expressed as the means (SDs). ^a^ 1 mL of 10^9^ CFU/kg body weight was injected intravenously. Significantly differed values are indicated with different capital letters.

**Table 3:** Counts of viable MRSA in blood samples.

Group/Number	Inoculation ^a^	Survival Rate (Day PI ^b^)	MRSA Count/mL at Different Blood Sampling Time-Points (Days)							
			−10 ^c^	1	2	3	4	5	6	7
1/5	MRSA	0	0 ^A^	2260 ^A^ ± 976	1780 ^A^ ± 325	2180 ^A^ ± 325	2250 ^A^	2140 ^A^	1910 ^A^	2610 ^A^
2/5	MRSA + vancomycin (0.5 mg/kg)	80	0 ^A^	430 ^C^ ± 303	147 ^C^ ± 96	20 ^F^ ± 11	4 ^E^ ± 2	2 ^E^ ± 1	ND ^E^	ND ^E^
3/6	MRSA + Epi-1 (1.5 mg/kg)	83.3	0 ^A^	393 ^C^ ± 57	220 ^C^ ± 65	12 ^F^ ± 4	ND ^E^	ND ^E^	ND ^E^	ND ^E^
4/6	MRSA + Epi-1 (2.5 mg/kg)	100	0 ^A^	6 ^F^ ± 2	ND ^F^	ND ^F^	ND ^E^	ND ^E^	ND ^E^	ND ^E^
5/6	MRSA + Epi-1 (1.5 mg/kg) + vancomycin (0.5 mg/kg)	100	0 ^A^	95 ^D^ ± 36	11 ^F^ ± 19	17 ^F^ ± 4	ND ^E^	ND ^E^	ND ^E^	ND ^E^

^a^ 1 mL of 10^9^ CFU/kg body weight was injected intravenously. ^b^ PI, post inoculation. ^c^ “–10” indicates 10 minutes prior to inoculation. ND, no detection. Sampling time-points for bacteriology were –10 min and 1, 2, 3, 4, 5, 6, and 7 days, as indicated in Table 1. Significantly different values are indicated with different letters.

**Table 4:** Viable counts of MRSA in heart, lung, liver, and kidney.

Group/Number	Inoculation ^a^	Pig Death (Day PI ^b^)	MRSA Count/g at Different Organs			
			Heart	Lung	Liver	Kidney
1/5	MRSA	1, 2, 4, 7	310,000 ^A^ ± 12000	41,000 ^A^ ± 2930	45,000 ^A^ ± 6100	150,000 ^A^ ± 30000
2/5	MRSA + vancomycin (0.5 mg/kg)	1, 7	110,000 ^BC^ ± 9200	40,000 ^AB^ ± 12,000	14,000 ^C^ ± 960	23,000 ^C^ ± 1100
3/6	MRSA + Epi-1 (1.5 mg/kg)	2, 7	220,000 ^B^ ± 15,000	32,000 ^BC^ ± 6000	20,000 ^BC^ ± 1300	13,000 ^D^ ± 6500
4/6	MRSA + Epi-1 (2.5 mg/kg)	7	ND ^E^	ND ^E^	ND ^E^	ND ^E^
5/6	MRSA + Epi-1 (1.5 mg/kg) + vancomycin (0.5 mg/kg)	7	ND ^E^	ND ^E^	ND ^E^	ND ^E^

^a^ 1 mL of 10^9^ CFU/kg body weight was injected intravenously. ^b^ PI, post inoculation. ND, not detectable. Bacterial counts were performed on tissues from heart valve, left diaphragm lobe of lung, left lateral lobe of liver, and left kidney from each pig following death from infection or after euthanasia. The samples were stored at 4 °C for 12 h before processing. Sample tissue was aseptically removed and cut into small pieces. One gram of tissue was homogenized with a stomacher blender in 9 mL of sterile isotonic saline, and ten-fold dilutions were prepared. From each dilution, 10 µL was cultured on mannitol salt agar with oxacillin (2 µg/mL) medium at 37 °C for 48 h prior to counting the colonies. Significantly different values are indicated with different letters.

**Table 5:** Histopathology in major organs.

Organ	Clinical Sign (t/n ^a^)	Group				
		MRSA	MRSA + Vancomycin (0.5 mg/kg)	MRSA + Epi-1 (1.5 mg/kg)	MRSA + Epi-1 (2.5 mg/kg)	MRSA + Epi-1 (1.5 mg/kg) + Vancomycin (0.5 mg/kg)
Heart	Infective endocarditis	5/5	0/5	0/5	0/5	0/5
Lung	Inflammation, pulmonary edema	5/5	2/5	4/5	2/5	2/5
Liver	Liver abscess	5/5	1/5	1/5	0/5	0/5
Kidney	Cystic tubules, cortical	5/5	0/5	0/5	0/5	0/5

^a^ Number of animals with clinical signs/total number of examined animals.

## Supplementary Materials

The following are available online at https://www.mdpi.com/1660-3397/17/12/693/s1, Supplementary Figure S1: Pharmacokinetics of vancomycin in pigs after intravenous administration. Femoral vein of pig was i.v. infused with 0.5 mg/kg of vancomycin or 1.5 mg/kg Epi-1 plus 0.5 mg/kg vancomycin in PBS. Blood samples were withdrawn from the carotid artery at 0, 10, 30, and 60 min, and then at 60-min intervals up to 360 min. Serum was obtained from the blood samples, and the concentration of vancomycin was determined by liquid chromatography-tandem mass spectrometry (LC-MS-MS). The data are shown as means ± SEMs, and are representative of two independent experiments. Supplementary Table S1: A critical assessment of sepsis and evaluation of vital signs, including temperature and blood pressure. Supplementary Video: Survival and activity of pigs at 1, 2, and 7 days post-infection with MRSA and treated with antibiotic and Epi-1. Five or six pigs were used in each group. Five pigs were inoculated with the MRSA strain (1 × 10^9^ CFU/kg); five pigs were treated with vancomycin (0.5 mg/kg) in sterile isotonic saline; six pigs were treated with Epi-1 (1.5 mg/kg) in sterile isotonic saline; six pigs were treated with Epi-1 (2.5 mg/kg) in sterile isotonic saline, and six pigs were treated with Epi-1 (1.5 mg/kg) plus vancomycin (0.5 mg/kg) in sterile isotonic saline 30 min after MRSA infection. Supplementary Table S2: Details of the primers used in Staphylococcus aureus gene-specific multiplex PCR.
