# Supplementary material for: Epinecidin-1 Protects against Methicillin Resistant Staphylococcus aureus Infection and Sepsis in Pyemia Pigs
Source: Mar Drugs. 2019 Dec 9;17(12):693. doi: 10.3390/md17120693 (PMC6950563; doi:10.3390/md17120693)
Supplement: Supplementary file 1 [file marinedrugs-17-00693-s001.zip › Supplementary/Supplementary.pdf]

Supplementary Table 1. A sepsis critical assessment evaluation of vital signs, including temperature and blood pressure.

Number	Inoculation[a]	Critical assessment	Timepoints (day)				
			−10[b]	1	2	3	4
		Temperature (°C)	36.5	37.7	38.3	38.6	38.1
1	MRSA	Systolic blood pressure (mmHg)	110	82	88	62	78
		Bacteremia (CFU/mL)	0	1135±127	879±54	745±87	1412±182
		Temperature (°C)	35.9	38.1	39.1	38.6	38.4
2	MRSA	Systolic blood pressure (mmHg)	92	93	82	78	82
		Bacteremia (CFU/mL)	0	931±357	635±42	955±184	844±127
		Temperature (°C)	36.1	37.7	38.6	37.9	38.1
3	MRSA	Systolic blood pressure (mmHg)	89	91	88	59	77
		Bacteremia (CFU/mL)	0	513±187	874±23	791±84	531±287

a 1 mL of 10^9 CFU/kg body weight was injected intravenously

b "-10" indicates 10 min prior to inoculation

Timepoints for bacteriology were -10 min, 1, 2, 3, and 4 day as indicated in supplementary Table 2.

Supplementary Table 2. Details of the primers used in *Staphylococcus aureus* gene-specific multiplex PCR.

**Primer information**
**Eta-F'**: GCAGGTGTTGATTTAGCATT
**Eta-R'**: AGATGTCCCTATTTTTGCTG
**mecA-F'**: ACTGCTATCCACCCTCAAAC
**mecA-R'**: CTGGTGAAGTTGTAATCTGG
**femA-F'**: AAAAAAGCACATAACAAGCG
**femA-R'**: GATAAAGAAGAAACCAGCAG
**Etb-f'**: GATAAAGAAGAAACCAGCAG
**Etb-r'**: GTTTTTGGCTGCTTCTCTTG

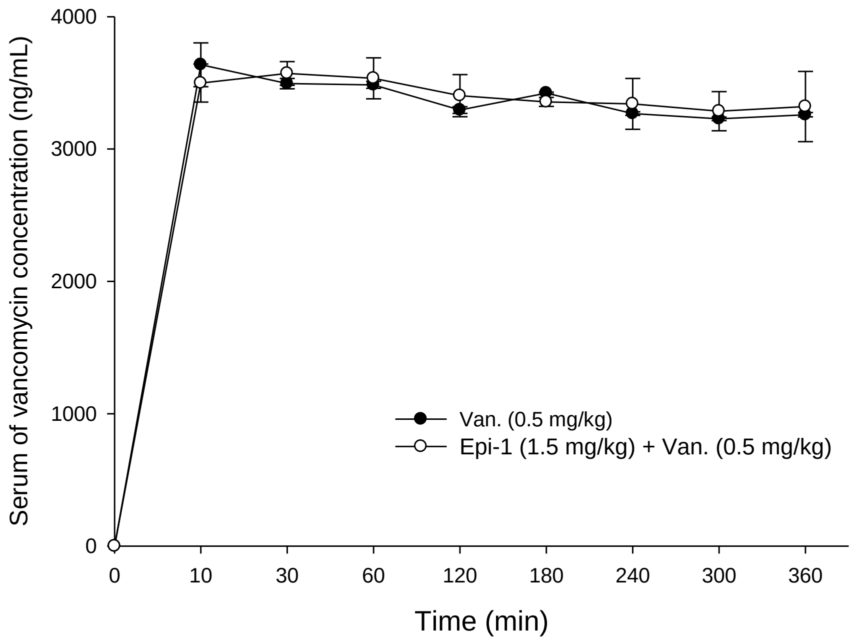

Supplementary Figure 1. Pharmacokinetics of vancomycin in pigs after intravenous administration. Femoral vein of pig was i.v. infused with 0.5 mg/kg of vancomycin or 1.5 mg/kg Epi-1 plus 0.5 mg/kg vancomycin in PBS. Blood samples were withdrawn from the carotid artery at 0, 10, 30, and 60 min, and then at 60-min intervals up to 360 min. Serum was obtained from the blood samples, and the concentration of vancomycin was determined by liquid chromatography-tandem mass spectrometry (LC-MS-MS). The data are shown as means ± SEMs, and are representative of two independent experiments.
